# Experimental characterization of a fully polarimetric pulsed terahertz spectroscopy system

**DOI:** 10.3389/fphy.2024.1317576

**Published:** 2024-02-02

**Authors:** Nikita Gurjar, Morgan E. Ware, Magda El-Shenawee

**Affiliations:** 1 Department of Electrical Engineering and Computer Science, University of Arkansas, Fayetteville, AR, United States; 2 Material Science and Engineering, University of Arkansas, Fayetteville, AR, United States

**Keywords:** terahertz imaging, spectroscopy, wave polarimetry, x-cut crystal, birefringent waveplate

## Abstract

A terahertz time domain pulsed spectroscopy system is modified to provide fully polarimetric radiation and analysis. The operation of this polarimetry system is characterized using a birefringent, x-cut quartz crystal. The modification is based on rotating the photoconductive antennas such that both the emitted and detected polarizations are out of the plane of incidence. Subsequently, broadband wire grid polarizers are used to select the incident and detected direction of linear polarization to be either parallel with (vertical) or perpendicular to (horizontal) the plane of incidence with the sample surface. The experiments are conducted in both transmission and reflection. Depending on the frequency, the phase retardation of the incoming electric field components along the two perpendicular optical axes of the quartz crystal changes differently. This results in the polarization of the light exiting the crystal changing with frequency. As a result, multiple frequencies are identified where the crystal behaves as a near ideal quarter-, half-, or full-wave retarder. Additionally, due to the time-domain nature of the experiment, transmitted and reflected electric fields are measured after multiple consecutive reflections within the crystal. This leads to a further, complex control over the final polarization state of the signal. Finally, images of a resolution standard are obtained demonstrating the characteristics of the polarimetry system.

## 1 Introduction

Terahertz radiation (THz) has emerged as a non-ionizing form of radiation with much more potential for detecting small features than radio, microwave, or ultrasound, while at the same time exhibiting much higher penetration into mammalian tissue than visible light. In fact, THz waves possess extremely sensitive absorption and spectral signatures to various tissue conditions [1, 2]. However, a technique for the general manipulation of the state of polarization of THz light has not been widely developed and utilized within the field of medical imaging. In this paper, we demonstrate one technique based on optical retarders and characterize its effectiveness through polarization dependent THz spectroscopy and imaging [3].

An optical retarder is generally an element that slows one component of polarization with respect to another perpendicular component [4]. It is defined by the ordinary and extraordinary indices of refraction (*n*_o_ and *n*_e_), which govern light transmission with polarizations along the ordinary and extraordinary axes, respectively [5]. These result in a difference in the velocity of waves polarized along each direction and an associated phase shift between the ordinary and extraordinary components of the transmitted light [6].

Quartz is a well-known birefringent material, exhibiting ordinary and extraordinary axes [7, 8]. At the same time, quartz is highly transparent to THz radiation, establishing it as a good source material for the fabrication of optical retarders in the THz regime, and it has been demonstrated as such [9, 10], with several designs for achromatic waveplates [7, 11, 12]. A parameterization of the ordinary and extraordinary indices over a wide wavelength range is given in [13]. To use these crystals as retarders, they must be cut and polished parallel with the optic axis, such that the optic axis lies in the plane of the surface. This is the so-called x-cut of the quartz crystal or the ($10\bar{1}0$) Miller-Bravais lattice plane [14, 15]. With more of a focus on the use of quartz birefringent waveplates for the THz characterization of other materials through spectroscopy, there have been several demonstrations of technological implementations of these devices in spectroscopic systems. Many of these examples have focused on complex designs of stacks of multiple waveplates [11].

For the THz regime, much of the technology for polarization control was adopted from microwave and millimeter wave systems. For example, Huang et al. used appropriately sized waveguide horn antenna to create linearly polarized radiation at up to 0.3 THz [16]. With the addition of 3D printed phase matched lenses, they were able to both focus and introduce either left or right circularly polarized beams. Subsequently, polarization measurements were simply made by rotating the emission and or detection optics by either 45° or 90° as needed. This technique is limited to low, single frequency measurements.

A more general technique for manipulating polarization in the THz regime was to exploit the generation of the THz beam itself through the nonlinear electro optic effect [17]. In this case if a short pulse of optical frequency light is incident on a nonlinear electro optic crystal like ZnTe, broadband THz radiation is generated, possibly with frequencies around the range of 0.1–5 THz. Due to the non-isotropic nature of zinc blende crystals like ZnTe, the orientation of the crystal can be exploited to control the linear polarization of the emitted THz radiation. If a (111) ZnTe crystal was used, the rotation of the emitted linear polarization appears at an angle twice that of the rotation of the direction of linear polarization of the incident optical pulse. Therefore, controlling the linear polarization of the incident light would directly control the polarization of the emitted THz pulse. Furthermore [17], described using achromatic-prism quarter-wave retarders made from either silicon or the plastic ZEONEX to convert the linear polarization of the THz pulse to circular and back. While this technique is relatively versatile, the described Fresnel rhomb structures were bulky and difficult to keep aligned. At the same time the linear polarization of the source was dependent on using the electro-optic effect in ZnTe-like crystals. However, sensitivity and versatility has been increased in the form of more recent spinning electro optic measurements, where the detector crystal was rotated at some rate, while the measurement was performed in phase with the rotations [18].

Single frequency THz systems based on far infrared laser emission are inherently linearly polarized such as CO_2_ pumping of formic acid (HCOOH) resulting in 0.584 THz radiation [19]. Polarization sensitive measurements were subsequently facilitated by ideally matched, single frequency quartz half- and quarter-waveplates. While this type of technique does generate high brightness THz beams, the single frequency measurements are limiting, and the optical setup is complicated and expensive.

Traditional THz-TDS spectroscopy systems, which use bowtie-like emitters are inherently polarized, however controlling this polarization and determining depolarization effects of measured samples is still a challenge. One recent novel approach to solving this was through the use of decoupled, simultaneous, crossed emitters [20]. Here, crossed bowtie antennas were bridged by InP nanowires as the high mobility semiconductor platform. In this case the emission and detection from these devices were not only polarized to a higher degree, the decoupled-crossed configuration allowed for simultaneous measurements of both orthogonal linear polarizations. This technique is fast and sensitive, but the fabrication of the nanowire antennas was extremely labor intensive, while their use in a THz-TDS system requires extra electronic connections.

Our existing THz time-domain pulse spectroscopy (TPS) system uses voltage-biased bow-tie photoconductive antennas for the generation of time domain THz pulses after excitation from a femtosecond Ti: Sapphire laser emitting 780 nm pulses. The system is manufactured by TeraView Ltd. in UK as described in [21–26]. The designs of the emitter and detector photoconductive antennas (PCAs) are proprietary information of the manufacturer. The Fourier Transform of the time-domain pulse provides a frequency spectrum from ~0.1 to 4 THz. Both the emitting and detecting antennas are originally oriented in one direction (e.g., parallel to the plane of sample incidence). This orientation only provides a co-polarized THz signal (e.g., vertical-vertical polarization) with the electric field parallel to the plane of incidence. More details will be provided in the next Section. In the present work, we will present a modification to the standard spectroscopic system of limited polarization capabilities, such that fully polarimetric signals can be obtained and characterized. The modified system includes all four combinations of in and out of the plane of incidence with the sample, i.e., vertical, and horizontal linear polarizations, respectively. Furthermore, we present experimental results of the interaction of THz polarimetry radiation with the quartz crystal in transmission and reflection modes. Spectroscopy and imaging results are demonstrated to further validate the system polarimetric characterization.

The manuscript is organized as follows; introduction in Section 1, materials and methods in Section 2, results in Section 3, conclusion in Section 4, and supplementing results in Appendix A.

## 2 Materials and methods

**FIGURE 1:**
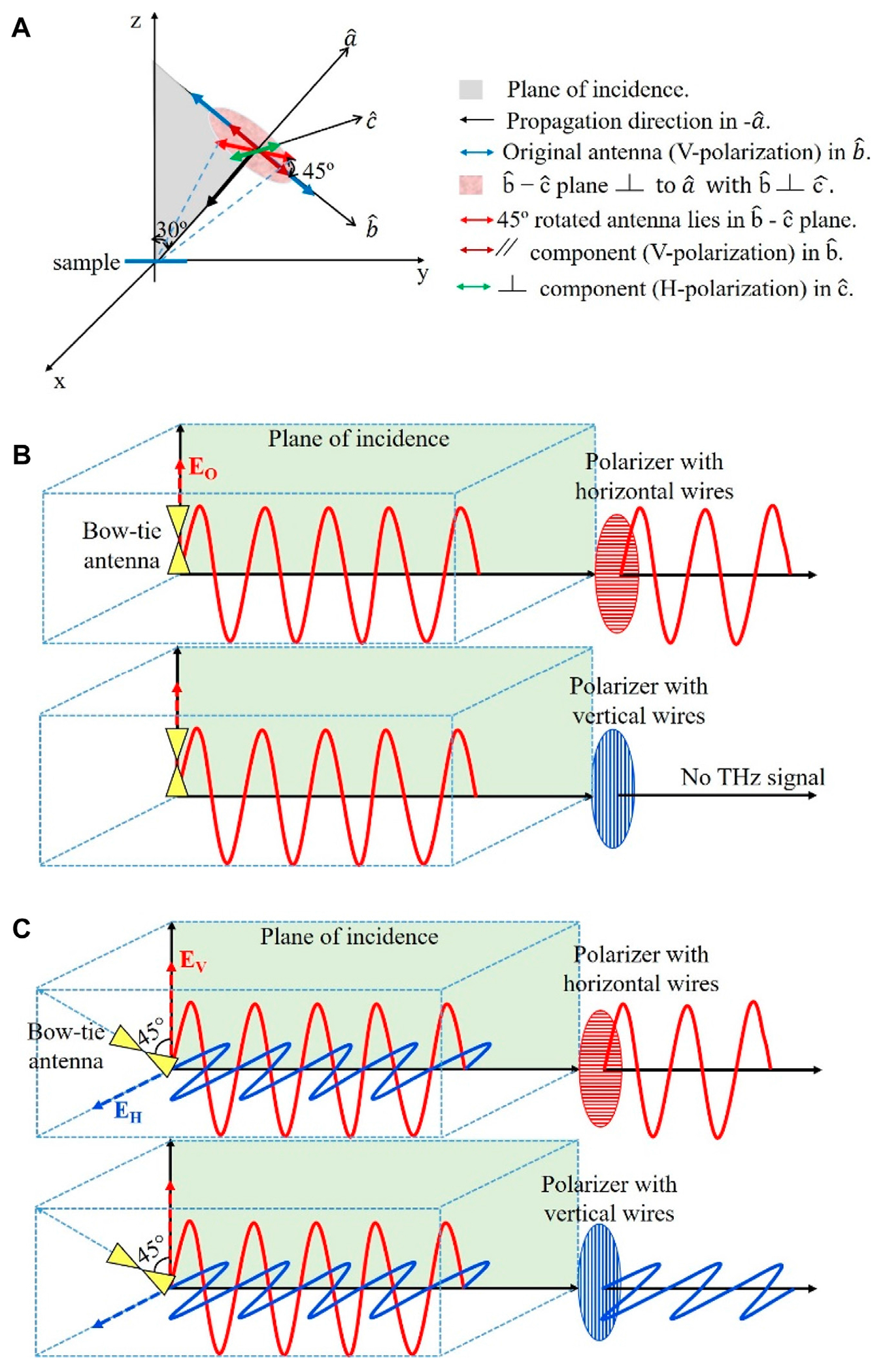
Sketch of THz signal polarization. **(A)** antenna rotation out of the plane of incidence, **(B)** signal before rotating the antennas, and **(C)** signal after rotating the antennas. The blue and red colors in B and C represent horizontal and vertical polarizations, respectively.

The THz heads of the system encapsulate the emitting and detecting antennas along with optics and biased voltage source. Bow-tie antennas, utilized in both the emitter and detector, are rotated by approximately 45° out of the plane of incidence maintaining the alignment with the exciting laser. A schematic diagram showing all the relevant components with respect to the sample plane of incidence is depicted in Figure 1A.

The existing system provides a single antenna in the emitter and a single antenna in the detector. Both antennas are aligned parallel to the plane of incidence as shown in Figure 1B. The directions of the wave propagation and the normal to the sample stayed unchanged. We rotated both the emitter and detector antennas to be out of the plane of incidence by 45°, which is equivalent to decomposing the electric field into two orthogonal directions: one is parallel to the plane of incidence (*E*_V_) and the other is perpendicular to the plane of incidence (*E*_H_) as shown in Figure 1C. The amplitude of each component is 1/√2 of the original field. This rotation provides a total of four emitted and detected electric fields. Wire grid polarizers are utilized to select which two electric fields will be emitted to and detected from the sample as shown in Figure 1C. We could have utilized 2 THz half-waveplates, one at the emitter and one at the detector, however these devices are narrow band and would have limited the use of the full spectrum of our system, which is 0.1 THz - 4 THz.

Each field component, *E*_V_ and *E*_H_, is selected using a freestanding wire grid polarizer (G50X20 from Microtech Instruments) in front of both the emitter and detector heads, with a resulting amplitude of 0.707 of the electric field in the original system of Figure 1B. Fixed 3D printed holders allowing the wire grid polarizers to rotate are utilized in the system. These polarizers offer a bandwidth of 0.05–3 THz and provide a transmission of ~97% when the wires are perpendicular with the incoming electric field (i.e., passing signal) and ~2% when the wires are parallel to it (i.e., blocking the signal) [27, 28].

**FIGURE 2:**
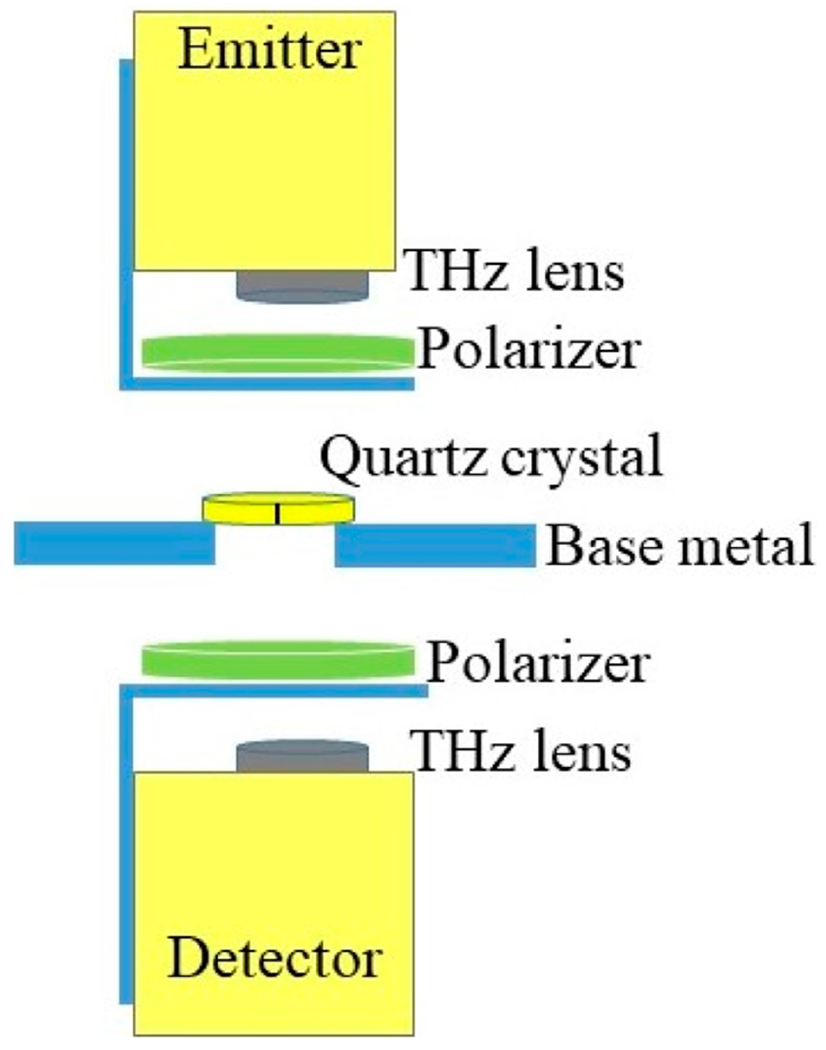
Transmission mode setup cross-section configuration of modified THz system. Fixed point spectroscopy data is collected.

Upon modifying the system hardware, it is crucial to experimentally examine and characterize the production of all four polarization combinations. Therefore, a 3.688 mm thick, x-cut quartz crystal with a diameter of 25.4 mm and both sides chemically polished (from Boston Piezo-Optics Inc.) is utilized as an optical retarder in Figure 2, to manipulate the state of the polarization. The x-cut quartz crystal is a frequency dependent device, i.e., it alters the incoming polarization depending on the frequency selective phase retardation between the field components along the two crystal axes (the extraordinary and the ordinary) [3, 5, 29, 30]. This relative phase retardation, τ_*r*_, between the field components is given by [4]:

(1) $$\tau_{r}=\frac{(n_{e}-n_{o})\;\omega \;L}{c}$$

Where *n_e_* and *n_o_* are the refractive indices of the extraordinary and ordinary crystal axes, respectively, *ω* is the angular frequency of light, *L* represents the physical path of the wave inside the crystal and *c* is the speed of light in vacuum. In this work, we obtained *n_e_* and *n_o_* using the data reported in [5]. When the relative phase retardation between the crystal axes is π/2, the crystal behaves as a quarter-waveplate, converting the incoming linear polarization into a circular polarization thereby giving us both the vertical and the horizontal field components. Similarly, when the relative phase retardation is π, the crystal behaves as a half-waveplate, rotating the incoming polarization from horizontal to vertical and *vice versa*. Furthermore, when the relative phase retardation is 2π, the crystal behaves as a full-waveplate and does not alter the incoming polarization [4]. Therefore, for a crystal of specific thickness, we can predict the polarization behavior at different frequencies. When characterizing the modified system experimentally using the x-cut quartz crystal, we can predict the detected polarizations throughout the bandwidth of the system from ~0.1 THz to ~4 THz.

It is important to place the crystal in the proper orientation such that its effect on the light polarization is predictable. To achieve this, the incoming electric field should be aligned exactly between the two crystal axes [4, 31]. To ensure the correct orientation throughout the experiment, a black line is marked at 45° between the two perpendicular crystal axes as shown in Figure 2. The figure demonstrates the fixed-point spectroscopy set-up of the modified system in transmission mode. The wire grid polarizers are closely positioned below the emitter and above the detector as shown in Figure 2.

### 2.1 Experimental setups

The time domain pulses are recorded at a single fixed point for spectroscopy and in a ~128 × 117 pixel array for imaging in the x-y plane using scanner motors with 200 μm step size. Upon extracting the time domain data from the system, it is converted to frequency spectra by FFT with 1024 sampling points. The data acquisition is performed in a nitrogen gas purged environment to avoid water signatures in the signal. For fixed point spectroscopy the raw time domain waveform signal is averaged 500 times over ~22 min. In image scanning, the signal is averaged 5 times for each pixel requiring ~2.5 h for each image for one polarization. This long acquisition time limits averaging in image scanning. Furthermore, averaging over frequency leads to losing the waveplate frequencies which are significantly important in the current work.

#### 2.1.1 Transmission

THz time domain pulses are recorded at a fixed point after transmission through the crystal as shown in Figure 2. The crystal is positioned on a metallic holder while the emitter and detector are placed above and below the holder, respectively. The holder is made of aluminum with a circular aperture at the center. The crystal is placed such that the incoming electric field polarization aligns with the black mark at 45° with respect to the crystal perpendicular axes. Upon manually steering the wire grid polarizers, as explained earlier, four different polarization conditions are produced and characterized: horizontal-horizontal, vertical-vertical, horizontal-vertical, and vertical-horizontal, i.e., HH, VV, HV, and VH, (the first and second words or letters represent the polarization of the received and incident signals, respectively [32]).

#### 2.1.2 Reflection

**FIGURE 3:**
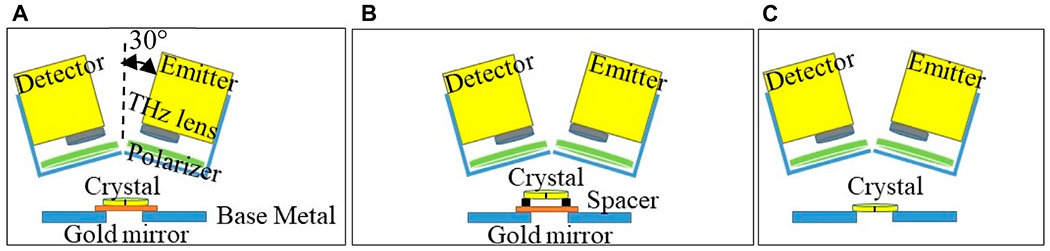
Configurations of modified THz time domain system in *reflection* mode setup for **(A)** quartz crystal positioned on a gold mirror, **(B)** quartz crystal positioned on 10 mm height spacer and a gold mirror, and **(C)** a stand-alone quartz crystal. Fixed point spectroscopy data was collected in each configuration.

The configuration of the reflection mode is shown in Figure 3. Here the emitter and detector heads are tilted 30° from the normal direction on the sample surface to avoid blocking the signals as shown in Figure 3. We present three different scenarios in this configuration: in Figure 3A the crystal is placed on a gold mirror (orange color), in Figure 3B the crystal is placed on a 10 mm spacer above a gold mirror, and in Figure 3C the crystal is placed with no back reflector. The data is collected at fixed point spectroscopy in each scenario. It is important to emphasize that the THz signal can be analyzed after reflecting off of multiple different surfaces of the crystal in this mode as shown in Figure 3.

## 3 Experimental results and discussion

### 3.1 Fixed point frequency spectroscopy in *transmission* and *reflection* modes

The frequency spectra of fixed points in transmission and reflection modes are collected. For every configuration, two frequency spectra plots are presented. For horizontal illumination, we collect both horizontal and vertical detector signals, i.e., HH and VH, and similarly for vertical illumination, i.e., VV and HV. In all figures in this Section, the co- and cross-polarized signals are presented in solid black and dashed red curves, respectively. Waveplate frequencies are defined and noted as those frequencies where the crystal behaves as quarter-, half-, or full-waveplates. In addition, all fixed-point spectra are unnormalized in this work.

**FIGURE 4:**
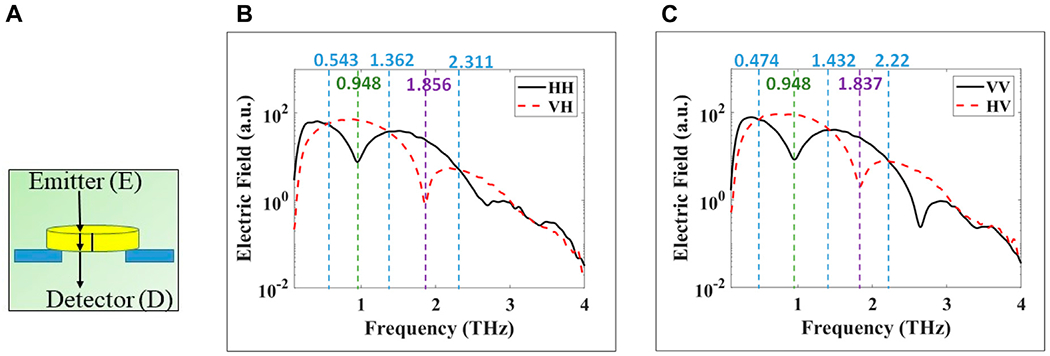
Experimental *transmission* at normal incidence of THz pulse through x-cut quartz crystal. Co- and cross-polarized are plotted in black and dashed-red, respectively. **(A)** propagation path in crystal, **(B)** horizontal polarization, and **(C)** vertical polarization. Dashed vertical blue lines indicate to waveplate frequencies of quarter-, green to half, or purple full-wave plate crystal behavior. The values of these frequencies are written in same colors at the top of the figure.

Figure 4A shows the signal propagation path through the x-cut quartz crystal in transmission. At normal incidence in transmission, *L* in Eq. 1 is the physical thickness of the crystal as shown in Figure 4A. Figures 4B,C show the resulting transmitted frequency spectra for horizontal and vertical illumination polarization, respectively. The frequencies marked by dashed-blue vertical lines mark the quarter-waveplate frequencies where the relative phase retardation is (2*n*+1)π/2 with *n* = 0, 1, 2, … , converting the incoming linear polarization to circular polarization. At these frequencies the curves of the co- and cross-polarizations intersect because circular polarization represents equal H and V electric field amplitudes in the detector. These frequencies are ~0.543, 1.362, and 2.311 THz for horizontal polarization in Figure 4B and ~0.474, 1.432, 2.22 THz for vertical polarization in Figure 4C. The frequency marked by a green vertical dashed line marks the half-waveplate frequencies where the relative phase retardation is π, converting between H and V polarizations. At this frequency, ~0.948 THz, the polarization is rotated by 90°, such that the cross-polarized signal is a maximum and the co-polarized signal is a minimum as shown in Figures 4B,C. Finally, the frequency marked by a purple dashed line marks the full-wave plate condition at ~1.856 and 1.837 THz where the phase retardation becomes 2π as shown in both Figures 4B,C, respectively. At this frequency, the signal passes without any polarization change. Other waveplate frequencies are seen across the bandwidth of the system from 0.1 to 4 THz, but we only marked a few of them in the figures. Some discrepancies are observed between the experimental waveplate frequencies and those obtained from Eq. 1 with (*n*_e_-*n*_o_) obtained from [5] as will be discussed in the Conclusion Section.

**FIGURE 5:**
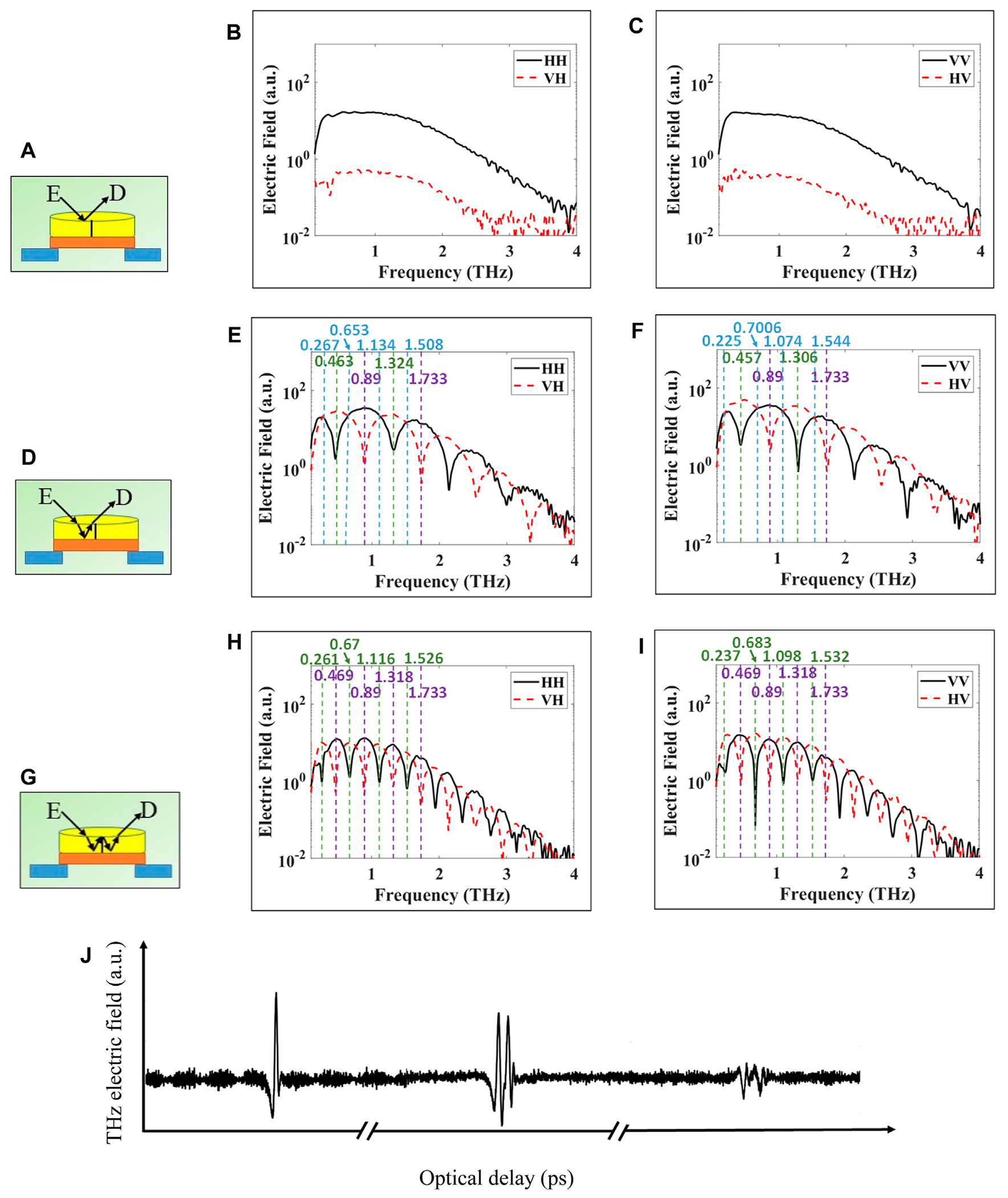
Experimental reflection of THz pulse from x-cut quartz crystal on top of gold mirror. **(A)**, **(D)**, **(G)** represent three different signal paths. THz polarimetry signals are obtained in time domain and transformed to frequency domain via FFT as shown in sub-figures. **(B)**, **(E)**, **(H)** horizontal polarization. **(C)**, **(F)**, **(I)** vertical polarization. Dashed vertical blue lines indicate to waveplate frequencies of quarter-, green to half-, or purple full-wave plate crystal behavior. The values of these frequencies are written in same colors at the top of figure except in Figures 5H and 5I where the frequencies of quarter-wave plates are omitted due to limited space. **(J)** Schematic diagram of three pulses associated with Figures 5A, 5D, and 5G, respectively.

Figures 5A, D, G depict the signal reflecting off of multiple different surfaces of the crystal. Figure 5J demonstrates three different time domain pulses, detected along the time delay axis in the system, associated with the configurations of Figures 5A, D, G, respectively. Each pulse is individually maximized by changing the height of the sample stage before recording. For the configuration in Figure 5A, the horizontal and vertical polarization results are shown in Figures 5B,C, respectively. In this case, the THz signal reflects from the crystal top surface leading to no alteration in the polarization of the incoming wave. The reflected horizontally polarized signal is detected as shown in Figure 5B solid black curve, while there is no reflected vertically polarized signal (red-dashed curve is insignificant). Similar results are shown in Figure 5C where the incoming and detected reflected signals have vertical polarization (black solid curve). There was no reflected horizontal polarization detected in this case either (red dashed curve).

In Figure 5D, the THz signal propagates inside the crystal and reflects from the back, enhanced by the gold mirror. Here, the wave travels a full round trip through the crystal, doubling the physical path length *L* in Eq. 1, and similarly doubling the total phase retardation at each frequency. The results of Figures 5E,F demonstrate nearly the same features as are found in transmission from Figures 4B,C, except that the frequencies are roughly half those found in transmission. Additionally, in reflection, higher order examples of the waveplates are now visible due to the increased effective path length. So, we find for the (2*n*+1)π/2 condition with *n* = 0, 1, 2, … multiple orders of quarter-waveplates (delineated by the blue dashed lines), for example, 0.267, 0.653, 1.134, and 1.508 THz for horizontal polarization in Figure 5E and 0.225, 0.7006, 1.074, and 1.544 THz for vertical polarization in Figure 5F. We also find the (2*n*+1)π condition for multiple orders of half-waveplates (delineated by the green dashed lines), for example, 0.463 and 1.324 for horizontal polarization in Figure 5E and 0.457 and 1.306 THz for vertical polarization in Figure 5F. Additionally, we find the 2*n*π condition for multiple orders of full-waveplates (delineated by purple dashed lines), for example, 0.89 and 1.733 THz for both polarizations in Figures 5E,F.

In Figure 5G, the signal can be considered as traveling four times through the crystal leading to four times the physical path length *L* to obtain the phase retardation, *τ_r_*. Again, in comparison with Figures 5E,F, we see the same features in Figures 5H,I at approximately half the frequency for each, with several additional orders of each waveplate visible. The values of the waveplate frequencies are written at the top of Figures 5H,I except for the quarter-waveplates frequencies that are omitted due to the limited space. It is also observed that all signal amplitudes in Figures 5H,I are smaller than those in Figures 5E,F due to the longer attenuating path inside the crystal material. Several waveplate frequencies are also seen across the bandwidth of the system from 0.1 to 4 THz, but we only marked a few of them in all figures in Figure 5. The results in other configuration scenarios of Figures 3B,C are tabulated in the Appendix and they follow similar trends and interpretations as in Figure 5.

### 3.2 Imaging results in transmission and reflection imaging

**FIGURE 6:**
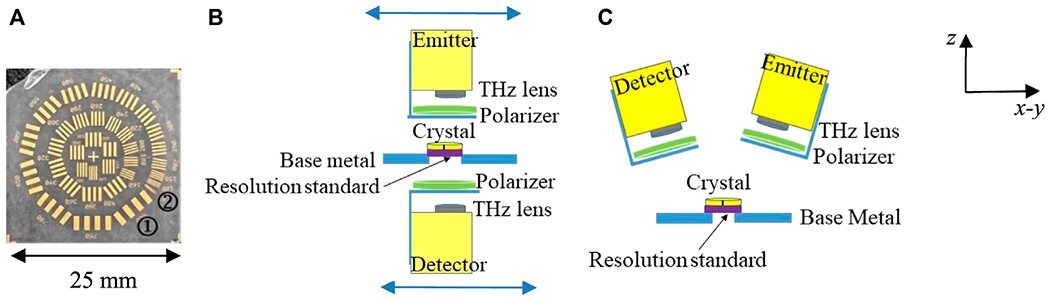
Imaging configuration of resolution standard positioned beneath the crystal. **(A)** photo of the square resolution standard with ① and ② mark the 800 μm and 20 μm resolution, respectively, **(B)** transmission mode, and **(C)** reflection mode. The horizontal blue double arrows indicate to the scanning process in *x*- and *y*-directions while the wave propagates in *z*-direction.

THz imaging of a square resolution standard, shown as an optical image in Figure 6A, is investigated at several waveplate frequencies determined above. Marks ① and ② in Figure 6A represent the 800 μm and 20 μm resolution, respectively. The resolution standard is positioned beneath the crystal as shown in Figure 6B for transmission and 6C for reflection. It is made of gold strips with varying spacing deposited on a polystyrene substrate. Each set of differently spaced strips are labeled with numbers describing the separation between strips in micrometers. No air gap is left between the crystal and the resolution standard. The data are acquired as a flyback pixelwise scanning in steps of 200 μm. Four polarization images are obtained: horizontal and vertical co- and cross-polarizations. The following consists of the presentation of images in both transmission and reflection modes taken with the linear polarizer in the standard four orientations, HH, VH, VV, and VH at frequencies corresponding to the various waveplates as determined above.

#### 3.2.1 Transmission imaging

**FIGURE 7:**
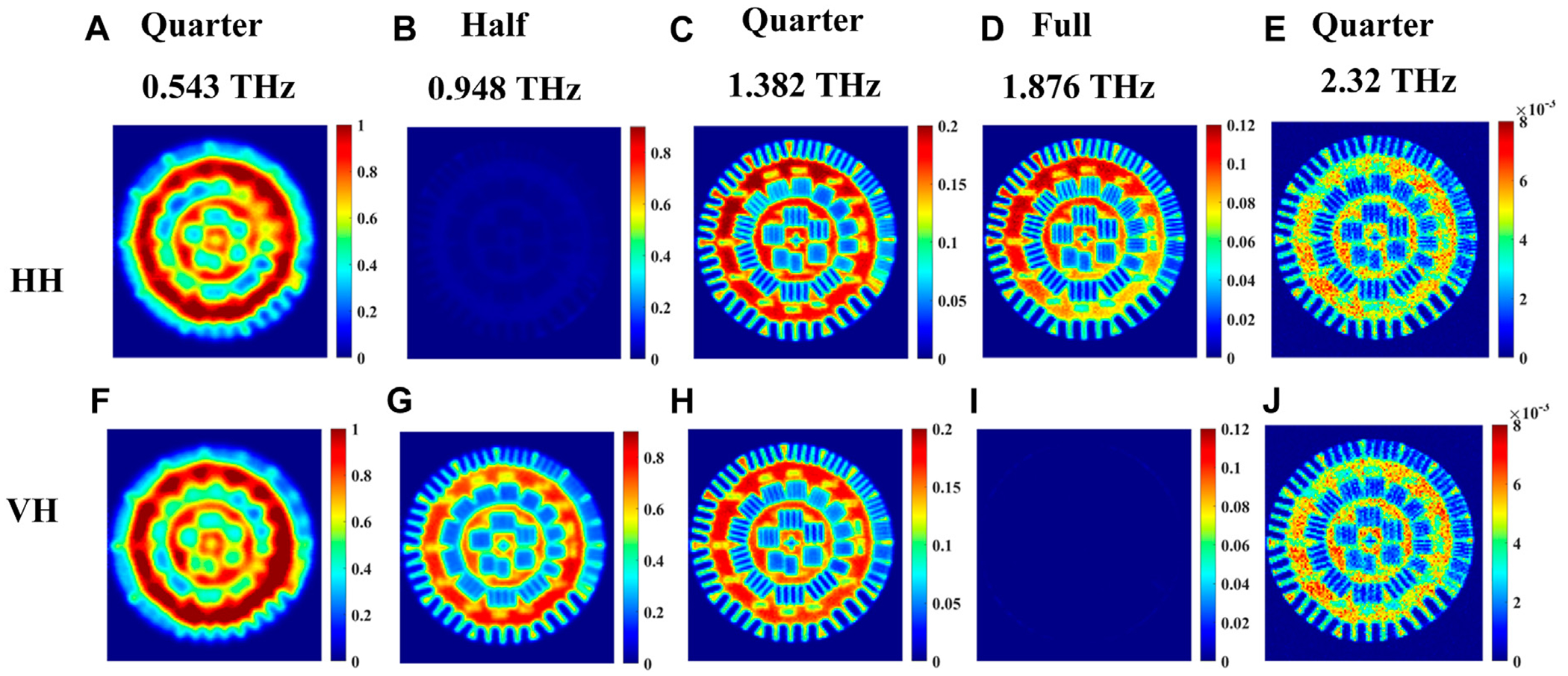
Transmission imaging of incoming *horizontal* polarization images at waveplate frequencies 0.543, 0.948, 1.382, 1.876, 2.32 THz of a resolution standard positioned beneath the crystal shown in Figure 2B. **(A–E)** represent the images for co-polarized HH signals, and **(F–J)** represent images of cross-polarized VH signals.

In transmission mode, the images are shown in Figures 7A–E for the co-polarized HH signals and in Figures 7F–J for the cross-polarized VH signals. All signals in transmission are normalized with respect to that of air. The propagation path of the signal inside the quartz crystal is the same as Figure 4A. Images are acquired at frequencies determined by single point measurements like in the previous Section. The HH and VH images at 0.543, 1.382, 2.32 THz are similar and therefore confirm that circularly polarized light was incident on the resolution standard from the quarter-waveplate at these frequencies, in Figures 7A, F; C, H; and E, J. However, it is obvious that the resolution increases with higher frequencies or shorter wavelength as would be expected. Ultimately, the highest resolution images in this set can be seen at 2.32 THz, due to the shorter wavelength of the radiation at this higher frequency. For the 0.948 THz images shown in Figure 7B, we find that the HH (co-polarized) image vanishes in comparison with the VH (cross-polarized) image in Figure 7G. This is confirmation that linearly polarized light, rotated 90° from the emission due to the half-waveplate at this frequency, is incident on the resolution standard. Conversely, the HH image (co-polarized) is visible for 1.876 THz in Figure 7D, while the VH (cross-polarized) image vanishes in Figure 7I. This is finally confirmation that the same linear polarization from the emitter is incident on the resolution standard and thus demonstrates a full-waveplate at this frequency.

**FIGURE 8:**
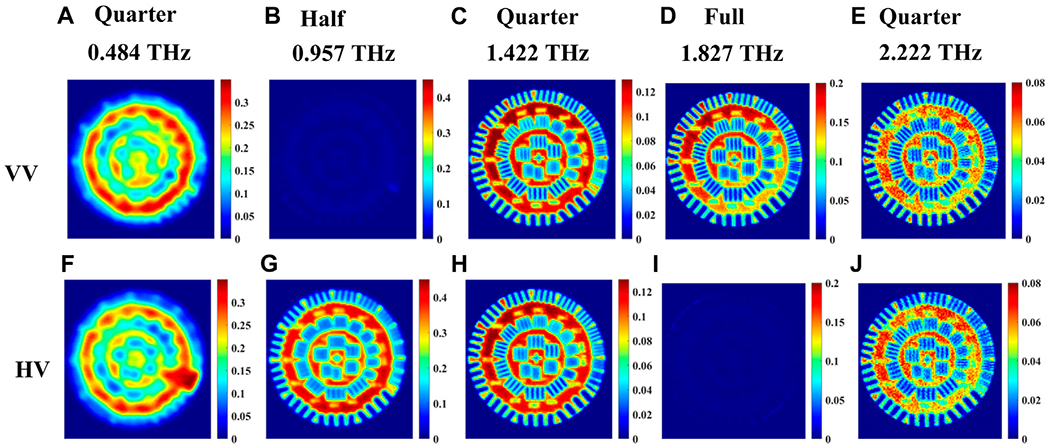
Transmission imaging of incoming *vertical* polarization images at waveplate frequencies 0.484, 0.957, 1.422, 1.827, and 2.222 THz of a resolution standard positioned beneath the crystal shown in Figure 2B. **(A–E)** represent the images for co-polarized VV signal, and **(F–J)** represent images of cross-polarized HV signal.

For the set of images where vertical polarization is fixed at the emitter, Figure 8 shows very similar responses as those in Figure 7, of horizontal polarization, both with slight shifts in the observed waveplate frequencies in Figure 4.

#### 3.2.2 Reflection imaging

**FIGURE 9:**
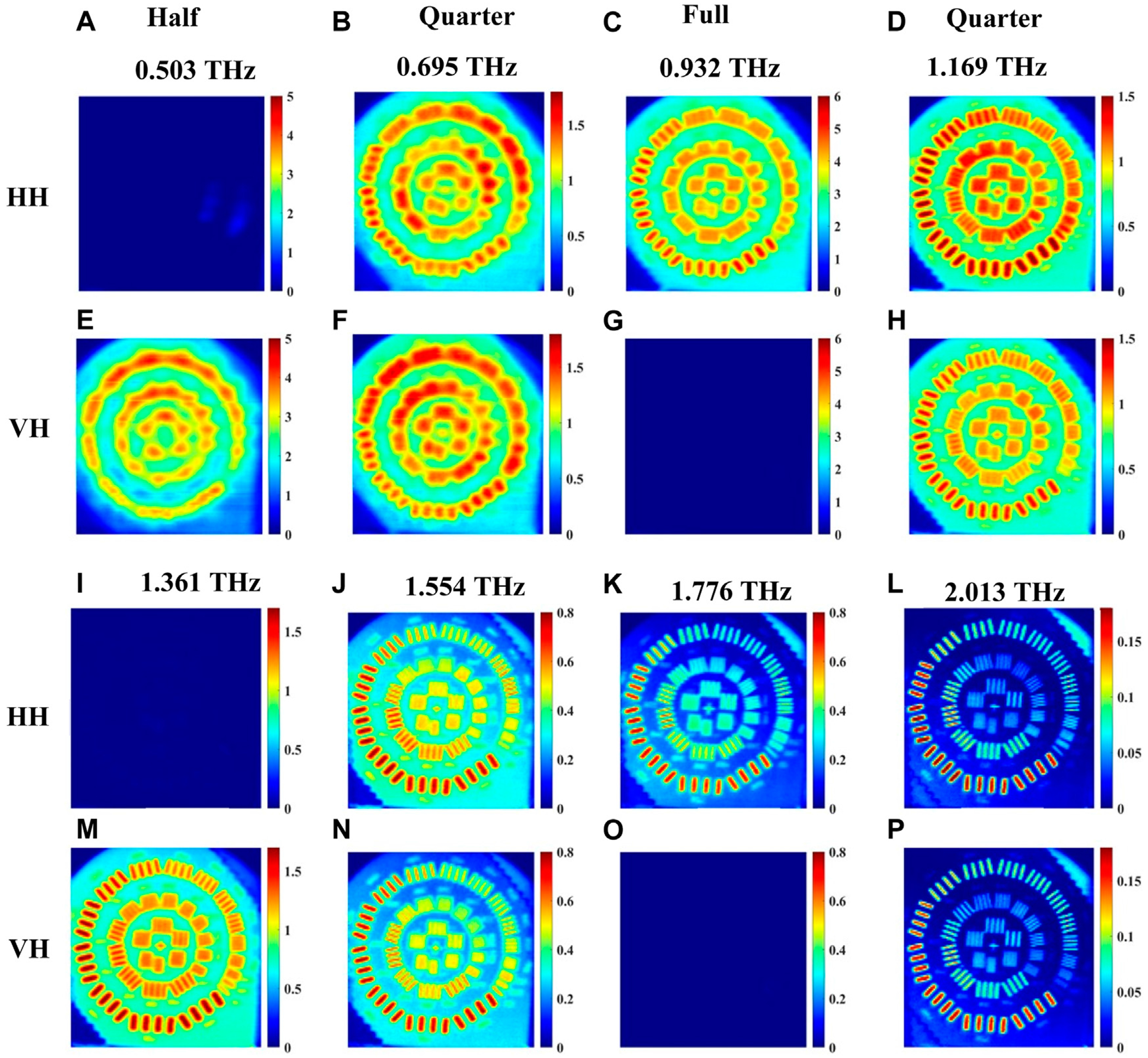
Reflection imaging of incoming *horizontal* polarization images at waveplate frequencies 0.503, 0.695, 0.932, 1.169, 1.361, 1.554, 1.776 and 2.013 THz of a resolution standard positioned beneath the crystal shown in Figure 6C. **(A–D)** and **(I–L)** represent the images for co-polarized HH signal, and **(E–H)** and **(M–P)** represent images of cross-polarized VH signal.

**FIGURE 10:**
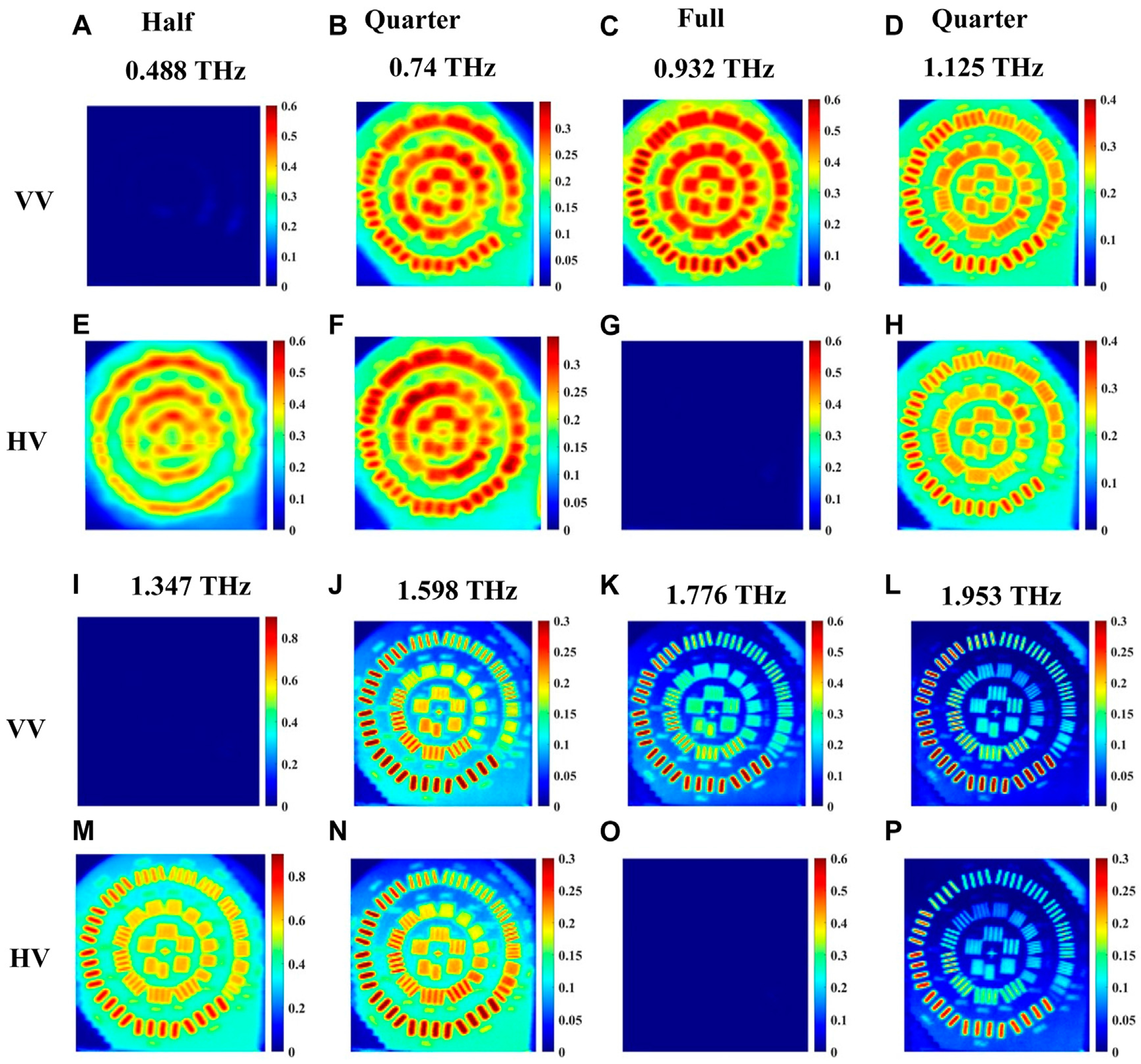
Reflection imaging of incoming *vertical* polarization images at waveplate frequencies 0.488, 0.74, 0.932, 1.125, 1.347, 1.598, 1.776 and 1.953 THz of a resolution standard positioned beneath the crystal shown in Figure 6C. **(A–D)** and **(I–L)** represent the images for co-polarized VV signal, **(E–H)** and **(M–P)** represent images of cross-polarized HV signal.

The experimental setup configuration for imaging in reflection mode is shown in Figure 6C. The images acquired in this configuration for horizontally polarized light from the emitter are shown in Figure 9. Figures 9A–D and 9I–9L are for co-polarized, HH, signals while Figures 9E–H and 9M–9P are for cross-polarized, VH, signals. Figure 10 presents similarly polarized images with the emitter light fixed as vertical polarization. All signals in reflection mode are normalized with respect to that of the gold mirror with no quartz crystal present. When the beam is above the gold strips of the resolution standard, the propagation path of the signal in the crystal is similar to that in Figure 5D. Images are acquired at waveplate frequencies obtained from a single point spectroscopy measurement on a gold strip following the procedure of the previous Section. Here, the raw data time domain pulses at each point within an image are averaged 5 times. In Figure 9, we selected eight waveplate frequencies where the crystal behaves as quarter-, half-, or full-waveplates, skipping the very first frequency to start and end with frequencies like transmission imaging in Figures 7, 8. It should be noted that relatively low resolution and increased noise level are observed at the low frequency of ~0.2 THz. Accordingly, we present eight images in each co- or cross-polarization as shown in Figures 9, 10, with the frequencies labeled in the figures. For example, in Figure 9, when the crystal behaves as a quarter waveplate, the images in Figures 9B,F at 0.695 THz look the same; at 1.169 THz the images in Figures 9D,H look the same; at 1.554 THz the images in Figures 9J,N look the same, and at 2.013 THz the images in Figures 9L,P look the same. When the crystal behaves as half waveplate, the co-polarized HH images in Figure 9A at 0.503 THz and 9I at 1.361 THz are not detected whereas the cross-polarized VH images at the same frequencies in Figures 9E,M, respectively, are bright. Conversely, when the crystal behaves as a full waveplate, the cross-polarized images VH in Figure 9G at 0.932 THz and 9O at 1.776 THz are not detected whereas the co-polarized HH images in Figures 9C,K, respectively, are bright. Similar observations are confirmed in Figure 10 for the vertical polarization images. As expected, again due to the shorter wavelength, the image resolution at higher frequencies, e.g., in Figure 9, from 1.169 THz to 2.103 THz, for any of the waveplate conditions is significantly better than the images at lower frequencies from 0.503 THz to 0.932 THz. Also, in Figure 10, the image resolution in the frequency range from 1.125 THz to 1.953 THz is significantly better than that in the images from 0.488 THz to 0.932 THz.

Slight differences between the waveplate frequencies in horizontal and vertical polarizations are observed here, similar to the imaging results in the transmission mode configuration. However, in the same manner as with the transmission images, we find that the details of the images, which should be the result of varying the retardance due to the quartz crystal, are precisely predicted by this basic understanding. At the same time, the resolution of the images improves very noticeably as the frequency increases, decreasing the wavelength of light over which the image is acquired.

## 4 Conclusion

The results in this paper demonstrate a method to obtain a fully polarimetric THz TPS characterization from an existing single polarization system by rotating the emitter and detector photoconductive antennas out of the plane of incidence by 45°. Each antenna then provides roughly equal contributions of horizontally and vertically polarized electric fields and upon positioning two wire grid polarizers, one in front of the emitter and the other in front of the detector, a fully polarized system is achieved. Detection of all four polarization combinations (HH, VV, VH, and HV) is demonstrated.

This polarimeter system is subsequently tested by observing the behavior of an x-cut quartz crystal as quarter, half, and full waveplates. This birefringent crystal alters the polarization or ellipticity of the incoming signal depending on the phase retardation between the two electric field components along the two crystal axes. The interactions of the THz signal with the crystal were investigated in several configurations in both transmission and reflection modes. Each configuration represents a different propagation path of the signal through the crystal. These results demonstrate the behavior of the crystal as each of a quarter-, half-, and full-waveplate depending on the frequency of the transmitted or reflected light. These waveplate frequencies are shown through the results of fixed-point spectroscopy. For frequencies where the crystal behaves as a quarter-waveplate, the spectra of co- and cross-polarized curves intersect indicating circular polarization. For frequencies where the crystal behaves as a half-waveplate or full wave plate, a dip appeared in the co-polarized and cross-polarized spectral curves, respectively. Furthermore, the interactions of the incoming signal with a resolution standard positioned immediately following the crystal are investigated and four different polarization images of the resolution standard are obtained at each waveplate frequency. These reproduce the potential for polarimetric characterization in a full imaging configuration, while allowing for increasing the resolution by utilizing the same type waveplate at higher frequencies. This increased resolution is a generally accepted phenomenon in optics resulting from the increased resolving power and decreased diffraction limited spot size of shorter wavelength light.

Upon comparing the propagation paths of single and double reflections, higher order waveplate conditions are found as should be expected due to the increased physical path length within the quartz crystal. At the same time, the frequency where each waveplate is realized is roughly found to decrease by a factor of two, resulting from the double pass through the crystal.

These results highlight slight discrepancies in the frequencies where the waveplates are demonstrated, between the experimental realizations and those obtained using Eq. 1 and the index of refraction values in [5]. It is observed that the discrepancies between the experimental waveplate frequencies and the theoretical ones range from ~2% to ~10% in transmission fixed point spectroscopy in the results associated with Figure 4. Similarly, in reflection, the overall discrepancies range from ~0.3% to ~7% in the results associated with Figure 5D and from ~0.2% to ~11% in the results associated with Figure 5G. However, through the very low frequency range, between ~0.2 and 0.4 THz, where the noise is very high, there is found a much higher deviation of ~20%–25%. This decreases to less than ~11% between 0.4 and 0.65 THz. Finally, above 0.65 THz until the maximum range detectable, we found deviations below ~5%, with many below ~1%. Generally, it is understood that the very low frequencies are not as repeatable. This results from the FFT calculation used to determine the frequency spectra and the much longer delay line scans required to have higher accuracy for lower frequency. It should be noted that increasing the averaging number applied to the raw data time domain pulses leads to significant improvement in the frequency determination of the waveplate conditions.

To understand the relatively wide range of deviations found, we believe a tighter tolerance of the surface of the x-cut quartz crystal must be obtained. For the particular crystal used in this study, the surface roughness is likely on the order of 20–50 μm. Since the wavelength of 1 THz light is 300 μm, this introduces an uncertainty in the determination of the phase retardation of the order of 1% according to Eq. 1, depending on where the incident beam intersects the crystal. Additional deviation results from the increased physical path length within the crystal and non-zero incident angles, and possibly even the flexures of the crystal and the surrounding mounts and stages resulting from a variation in the temperature of the room. For the present study, these are uncontrolled, but future development of this technique could take advantage of these ideas.

The presented results demonstrated that the amplitude level of the vertical polarization is slightly higher than the horizontal polarization due to the manual 45° rotation of the antennas. The level of each dip in the spectral results were sensitive to the crystal orientation, which was also set by hand. In addition, the accuracy of the FFT of the experimental time domain pulses, even after interpolation, might not have provided precise frequency locations or amplitudes of these dips. It should be noted that the experiments of the H and V polarizations utilized a manual rotation of the wire grid polarizers as well, resulting in some additional uncertainty due to leakage of the unintended polarization.

On-going research, utilizing the polarimetric THz TPS system presented in this work, is in place to investigate the interaction of the co- and cross-polarized THz signal with inhomogeneous breast cancer tumors. Ultimately, we aim at increasing image contrast using polarimetry THz imaging and hence enhance the differentiation between cancerous and noncancerous tissue regions in excised breast tumors.

## Data Availability

The raw data supporting the conclusions of this article will be made available by the authors, without undue reservation.

## Appendix A

Fixed-point waveplate frequencies of Figures 3B,C are summarized in Table A1. No averaging is applied to the time domain raw signals in these cases.

**TABLE A1:** Waveplate frequencies of the crystal in reflection configurations (E for emitter and D for detector).
